# The close relationship between sudden loss of smell and COVID-19^☆^

**DOI:** 10.1016/j.bjorl.2020.05.002

**Published:** 2020-05-25

**Authors:** Lucia Joffily, Aluan Ungierowicz, Andrea Goldwasser David, Bruna Melo, César Leandro Terra Brito, Luciane Mello, Priscilla de Souza Campos dos Santos, Rogério Pezato

**Affiliations:** a Universidade Federal do Estado do Rio de Janeiro (UNIRIO), Hospital Universitário Gaffrée e Guinle, Rio de Janeiro, RJ, Brazil; b Universidade Federal do Rio de Janeiro (UFRJ), Hospital Universitário Clementino Fraga Filho (HUCFF), Rio de Janeiro, RJ, Brazil; c Hospital Regional de São José dos Campos (HRSJC), Serviço de Otorrinolaringologia, São José dos Campos, SP, Brazil; d Hospital Municipal Nossa Senhora do Loreto (HMNSL), Serviço Craniomaxilofacial, Rio de Janeiro, RJ, Brazil; e Santa Casa de Misericórdia do Rio de Janeiro, Serviço de Otorrinolaringologia, Rio de Janeiro, RJ, Brazil; f Hospital Federal da Lagoa (HFL), Rio de Janeiro, RJ, Brazil; g Universidade Federal de São Paulo (Unifesp), Escola Paulista de Medicina (EPM), Departamento de Otorrinolaringologia, São Paulo, SP, Brazil

**Keywords:** COVID-19, SARS-CoV-2, Anosmia, Hyposmia, COVID-19, SARS-CoV-2, Anosmia, Hiposmia

## Abstract

**Introduction:**

The real number of COVID-19 cases may be underestimated since several countries have difficulty offering laboratory tests for all the population. Therefore, finding a symptom with a high predictive value would help in diagnostic and isolation strategies.

**Objective:**

To correlate the sudden loss of the sense of smell in the context of the COVID-19 pandemic with results of diagnostic tests for COVID-19.

**Methods:**

This is a cross-sectional observational study. An online questionnaire was digitally addressed to 725 outpatients in Brazil who reported partial or total sudden loss of the sense of smell from March to April 2020.

**Results:**

Total or partial sudden loss of the sense of smell showed high positive predictive value for COVID-19 diagnosis, during the COVID-19 pandemic in Brazil (88.8%). There were no differences between groups tested positive and negative in regard to demographic and clinical characteristics such as presence of allergy, rhinitis, neither to olfactory recovery time.

**Conclusion:**

The identification of sudden loss of the sense of smell during COVID-19 pandemic may serve as a sentinel symptom and may be a warning to establish measures to prevent the transmission of the disease.

## Resumo

**Introdução:**

O número real de casos COVID-19 pode estar subestimado, pois vários países têm dificuldade em oferecer exames laboratoriais para toda a população. Portanto, encontrar um sintoma com alto valor preditivo ajudaria nas estratégias de diagnóstico e isolamento.

**Objetivo:**

Correlacionar a perda súbita do olfato no contexto da pandemia da COVID-19 com os resultados dos testes de diagnóstico da COVID-19.

**Método:**

Trata-se de um estudo observacional transversal. Um questionário on-line foi enviado digitalmente a 725 voluntários que apresentaram perda súbita parcial ou total do sentido de olfato de março a abril de 2020 no Brasil.

**Resultados:**

A perda súbita total ou parcial do sentido do olfato apresentou alto valor preditivo positivo para o diagnóstico de COVID-19, durante a pandemia de COVID-19 no Brasil (88,8%). Não houve diferenças entre os grupos positivos e negativos em relação às características demográficas e clínicas, como presença de alergia, rinite e tempo de recuperação olfativa.

**Conclusão:**

A identificação de perda súbita do olfato durante a pandemia de COVID-19 pode servir como sintoma sentinela e pode ser um alerta para estabelecer medidas para impedir a transmissão da doença.

## Introduction

On December 31st of 2019, the Chinese Center for Disease Control and Prevention (CDC China) and Wuhan City Health Authorities reported an outbreak of pneumonia of unknown cause. In January 2020, CDC China identified a new coronavirus in the lower respiratory tract samples of these patients and released its genome sequence. The World Health Organization (WHO) named the disease caused by the new coronavirus, COVID-19 and its severe acute respiratory syndrome, SARS-CoV-2.^1^

The first case in Latin America was confirmed on February 26th in São Paulo and since then Brazil has registered the highest number of cases in Latin America.^2^ On April 20th of 2020, there were 40,581 confirmed patients, 2575 deaths and a lethality rate of 6.3%. The Southeast region presents more than 50% of the cases.^3^

Coronavirus belongs to a virus family that manifests itself through different clinical presentations and has been responsible for important epidemics. This new coronavirus (SARS-CoV-2) is not the first to infect humans. The identified species of this family are varied and two stand out as responsible for epidemics with a major impact on global health: Middle Eastern Respiratory Syndrome (MERS-CoV) and Severe Acute Respiratory Syndrome (SARS-CoV).^4^, ^5^, ^6^

COVID-19 has also been having a major impact on health due to its higher degree of transmissibility, leading to a rapid worldwide dispersion. The new coronavirus has the ability to be transmitted by contact between people through respiratory droplets, aerosol or through contaminated vomiting.^7^ The main symptoms identified so far are: fever, cough, fatigue, myalgia, arthralgia and dyspnea, which can result in respiratory failure.^4^ Non-respiratory symptoms such as palpitations, abdominal pain, diarrhea, headache and dizziness may precede respiratory symptoms, or come in isolation.^1^ It rarely shows significant upper uirway (UA) involvement, but this is not the case for lower airways.^5^ It is known that the virus has as target cells that express receptors of the Angiotensin 2 Converting Enzyme (ACE2). ACE2 receptors are expressed predominantly by epithelial cells of the lung, intestine, kidney, heart and blood vessels, the former being the main organ affected by SARS-CoV-2.^8^ However, this does not seem to be the only route of virus entry into the cells, since the liver is quite affected, although it does not have many ACE2 receptors.^9^

The human brain also shows itself as a site with expression of ACE2 receptors, which seems to justify the neurotropism of SARS-COV-2 by the Central Nervous System (CNS). Studies corroborate the hypothesis of tropism of the coronavirus by the olfactory neuroepithelium and of neurological manifestations in confirmed cases of the disease.^10^, ^11^, ^12^

The increase in cases of sudden loss of the sense of smell (SLoS) noted in medical care during the COVID-19 pandemic motivated the present study. The physiological importance of olfaction in identifying environmental factors and potential threats is so important that the loss of the olfactory sense is related to a reduction in life expectancy, even in individuals without diagnosis of neurodegenerative disease such as Alzheimer's or Parkinson's disease.^13^ Olfactory disorder is a problem already described in most of the countries affected by COVID-19.^14^

The relationship between olfactory symptoms and viral UA infections is already well established.^15^ Previous studies show the existence of post-viral olfactory dysfunction with human rhinovirus, parainfluenza, coronavirus and Epstein–Barr virus.^16^ According to studies from the USA, Japan and Europe on hyposmia or anosmia etiologies, the post-viral cause corresponds in 18%–45% of cases.^4^ In cases of SARS-Cov-2, however, the complaint of anosmia is not usually accompanied by nasal obstruction, a common symptom in other UA infections.^4^, ^5^, ^11^, ^17^, ^18^, ^19^, ^20^

In developing countries like Brazil, COVID-19 testing is restricted to a small portion of patients due to the lack of availability of tests for the entire population. Thus, mild and even moderate cases are not being reported through positive laboratory evidence.

In addition to the shortage of tests at the beginning of this pandemic, the tests available so far have low sensitivity. Sensitivity is dynamic and depends on the time of illness in which the patient resides. Viral detection by RT-PCR of nasopharyngeal swab has a sensitivity of around 63%.^21^ Serology has a sensitivity of less than 40% in the first week of symptoms.^22^

Identifying a symptom highly suggestive of COVID-19 in an epidemic region with the application of a questionnaire can help in the early diagnosis and allow the implementation of social isolation measures, thus obtaining greater control of disease transmission.^23^ Some countries such as France, Germany and England have already adopted these measures when complaining of anosmia.^24^, ^25^, ^26^

## Objective

The objective of this work is to correlate the SLoS in the context of the COVID-19 epidemic with results of diagnostic tests for SARS-CoV-2.

## Methods

### Study design and data collection

A cross-sectional observational study with individuals who presented partial or total SLoS from March 2020 in Brazil.

We evaluated information collected on a data survey produced in Google Forms of patients between 18 and 90 years of age, complaining of SLoS during the COVID-19 epidemic. The questionnaire was sent to outpatients in social media groups, screening of offices, medical clinics and public and private hospitals.

Among the items to be answered were questions about demographic issues, associated symptoms, comorbidities and whether the patient had tested for COVID-19 or not. The methodology used for testing the cases could be RT-PCR and/or serology. For almost the entire research period, RT-PCR was the only test available in Brazil, so the majority of cases had done this kind of test for COVID-19.

All the individuals included in the study received and signed the Informed Consent Form (ICF) via electronic means. The results published here refer to data collected in the first weeks of the study, based on questionnaires, completed from 26th March to 11th April 2020, covering a 17 day period. The questionnaire was answered by the patients participating in the study themselves and sent back to the researchers with all the questions necessarily answered.

For the follow-up of the study, another questionnaire was sent by e-mail to each one of the participants, two weeks after receiving the first questionnaire, for a new collection of data on symptoms and test results for COVID-19.

Patients who did not sign the consent form, under 18 years or over 90 years of age and those who eventually answered the questionnaire but did not refer to having SLoS were excluded.

### Statistical analysis

The data generated in the study were analyzed in SPSS 18 (IBM Corporation, NY, USA), and R Core Team (2020). A language and environment for statistical computing, R Foundation for Statistical Computing, Vienna, Austria (https://www.R-project.org/). For baseline data, mean and Standard Deviations (SD) were used for normally distributed data and median and range for data that were not normally distributed. Categorical variables were expressed as counts and percentages; Pearson's chi-squared test and Fisher's Exact Test was used to evaluate between-group differences in two categorical variables. The Mann–Whitney *U* test was applied to evaluate the statistical differences between patient groups; *p*-values of less than 0.05 were considered significant.

## Results

### Demographic and clinical characteristics

**Figure 1:**
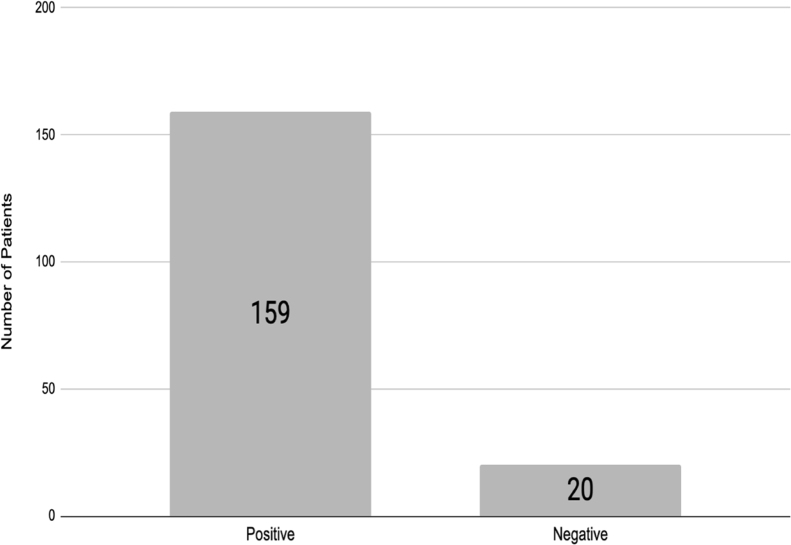
Frequency of test COVID-19 in sudden loss of sense of smell.

A total of 725 patients with SLoS who answered the questionnaire were included in the analysis. Of all participants, 546 (75.3%) could not perform any test for COVID-19 (not tested group). From the 179 (24.7%) who tested for COVID-19, 159 (88.8%) had positive results and 20 (11.2%) had negative results (Fig. 1). The clinical and demographic characteristics are shown in Table 1.

**Table 1:** Clinical and demographic variables associated with COVID-19 test in sudden loss of the sense of smell.

Characteristics	COVID-19 Test		*p*-value
	Negative	Positive	
	No. (%) *n* = 20	No. (%) *n* = 159	
*Age*			0.59^b^
Up to 39 years old	14 (70.0)	112 (70.4)	
40–59 years old	6 (30.0)	37 (23.3)	
60 years old and above	0 (0.0)	10 (6.3)	

*Gender*			0.74^a^
Male	5 (25.0)	50 (31.4)	
Female	15 (75.0)	109 (68.6)	

*Loss of the sense of smell*			0.33^b^
Complete loss	15 (75.0)	134 (84.3)	
Partial loss	5 (25.0)	25 (15.7)	

*Change in the taste*			0.38^b^
No	3 (15.0)	12 (7.5)	
Yes	17 (85.0)	147 (92.5)	

*Change in appetite*			0.40^b^
No	7 (35.0)	62 (39.0)	
Yes. Increased appetite	1 (5.0)	2 (1.3)	
Yes. Decreased appetite	12 (60.0)	95 (59.7)	

*Continuous use of nasal steroids*			0.47^b^
No	17 (85.0)	142 (89.3)	
Yes	3 (15.0)	17 (10.7)	

*Smoking*			0.08^b^
Never smoked	18 (90.0)	135 (84.9)	
Ex-smoker	0 (0.0)	19 (11.9)	
Smoker	2 (10.0)	5 (3.1)	

*Headache*			0.68^a^
No	4 (20.0)	43 (27.0)	
Yes	16 (80.0)	116 (73.0)	

*Cough*			0.95^a^
No	8 (40.0)	58 (36.5)	
Yes	12 (60.0)	101 (63.5)	

*Sore throat*			1.00^b^
No	14 (70.0)	107 (67.3)	
Yes	6 (30.0)	52 (32.7)	

*Shortness of breath*			0.22^b^
No	14 (70.0)	131 (82.4)	
Yes	6 (30.0)	28 (17.6)	

*Runny nose*			0.38^a^
No	15 (75.0)	99 (62.3)	
Yes	5 (25.0)	60 (37.7)	

*Nasal obstruction*			1.00^b^
No	16 (80.0)	126 (79.2)	
Yes	4 (20.0)	33 (20.8)	

a Pearson's Chi-squared Test.

b Fischer's Exact Test.

When we evaluated the age in the tested groups there was no statistical difference through them (*p* = 0.59). There was no statistically significant difference between positive and negative groups in regard of having partial or total SLoS (*p* = 0.33), neither in relation to the presence of other symptoms such as rhinorrhea (*p* = 0.38), shortness of breath (*p* = 0.22), cough (*p* = 0.95), sore throat (*p* = 1), nasal obstruction (*p* = 1), and headache (*p* = 0.68). Headache was the most prevalent symptom among patients regardless of the tested groups (73% in COVID-19 positive and 80% in COVID-19 negative).

Among tested patients, change of taste was highly associated in both groups: 17 (85%) in the negative group and 147 (92.5%) in the positive group, although there was no statistical difference between tested groups (*p* = 0.38). There was no statistical difference between negative and positive groups in relation to appetite alteration (*p* = 0.40), and about half of the patients had loss of appetite in both: 12 (60%) and 95 (59.7%) respectively.

Continuous use of nasal steroids showed no difference in the emergence of SLoS if partial or total in the two groups analyzed positive (*p* = 0.70) and negative COVID-19 (*p* = 1.00).

### Two-week follow-up

All participants who tested for SARS-CoV-2 (*n* = 179) were asked to answer a new questionnaire in two weeks after the first one in order to evaluate improvement of the sense of smell. At the beginning of the survey, 149 (83.2%) of those had total SLoS, being 134 (84.3%) COVID-19 positive group and 15 (75%) COVID-19 negative group. After two weeks, only 88 (55.3%) were reporting the symptom of loss of smell (partial or total) in the COVID-19 group, i.e., there was a recovery rate of 44.7% among individuals with SLoS after two weeks of follow-up in the group COVID-19 positive (Table 2). There was no significant difference in recovery after 2 week follow-up between tested groups, *p* = 0.17.

**Table 2:** Follow up of loss of smell in COVID-19 tested group.

	COVID 19 test	
	Negative	Positive
	No. (%) *n* = 20	No. (%) *n* = 159
*Onset*		
Partial Loss of Smell	5 (25%)	25 (15.7%)
Total Loss of Smell	15 (75%)	134 (84.3%)

*After two weeks*		
Complete Recovery	7 (35%)	71 (44.7%)
Partial Loss of Smell	8 (40%)	69 (43.4%)
Total Loss of Smell	5 (25%)	19 (11.9%)

Regardless of the tested groups, the history of allergic rhinitis did not influence the recovery from SLoS, with *p* = 0.76 in COVID-19 positive group, and *p* = 0.60 in COVID-19 negative group.

### Positive predictive value

SLoS showed high positive predictive value for COVID-19 diagnosis, during the Coronavirus Epidemic in Brazil 159 (88.8%) (Table 3).

**Table 3:** Loss of olfaction showed high Positive Predictive Value (PPV) for COVID-19 diagnosis.

Sudden loss of the sense of smell	COVID-19 test positive	COVID-19 test negative	PPV^a^
Positive	159	20	88.8%
Negative	0	0	

a PPV = 159/159 + 20.

## Discussion

During the COVID-19 pandemic, the availability of tests for laboratory confirmation of the disease was quite limited in relation to the demand for suspected cases in Brazil. Therefore, testing was prioritized for severe cases requiring hospitalization, underestimating the actual number of infected people and potentially overestimating the lethality of the disease. Despite the large number of patients in a short period of data collection, the majority (75.3%) of our participants couldn’t be tested for COVID-19.

Studies around the world indicate a correlation between olfactory dysfunction and SARS-CoV-2 infection.^4^, ^5^, ^11^, ^17^, ^18^, ^19^, ^20^ It is estimated that up to 2/3 of COVID-19 patients have SLoS and in most cases, as observed in the present study, patients described the olfactory dysfunction as sudden anosmia and less frequently hyposmia.^14^

In a not negligible number of patients, mainly paucissymptomatic, ageusia and anosmia may represent the first or only symptomatic manifestation of COVID-19.^5^, ^19^ In the present study 147 (92.5%) patients with SLoS tested positive for COVID-19 also presented a deficit of the taste.

The mechanism of olfaction impairment by COVID-19 is not clarified. One hypothesis is that SARS-CoV-2 would cause olfaction alteration by direct access and damage to the CNS through its penetration by the cribriform plate.^10^ Another hypothesis would be direct virus damage to olfactory cells and taste receptors. Glial cells, neurons and the oral cavity present ACE-2 receptors that seem to be the mechanism of cell invasion by the virus. Eliezer et al. published a case report of COVID-19 in which a 40 year-old woman sought medical attention for anosmia without nasal obstruction. The images obtained by CT and MRI showed inflammatory signs on the olfactory cleft, without alteration of the olfactory bulb. The possibility of a change in the volume of the olfactory bulb being very subtle at this stage of the disease could justify the absence of its alteration by MRI.^20^

Another aspect that must be mentioned is the number of individuals who had SLoS in such a short time. Potter et al. published a retrospective study that analyzed 587 patients with post-viral anosmia during one year of follow-up.^15^ Interestingly, in the present study we collected data from 725 patients with SLoS in just two weeks at the time of this data analysis. This sudden and surprising increase in the frequency of individuals with SLoS during the COVID-19 pandemic, makes us immediately suspicious that it is due to this type of coronavirus.

These preliminary results are in accordance with data produced by the French ENT Society.^22^ In our analysis, a high positive predictive value for the symptom of SLoS was observed in the group testing positive for COVID-19. Therefore, we think SLoS with or without nasal obstruction is a strong predictor of SARS-CoV-2 infection in the context of an epidemic. This data may become relevant when there is a shortage of laboratory tests for COVID-19. With this in mind, patients with SLoS with or without other symptoms may be classified as “strongly suspected cases”, allowing early implementation of measures to prevent transmission of the disease.

Other work carried out in the United Kingdom has established that the combination of anosmia, fever, fatigue, persistent cough, diarrhea, abdominal pain and loss of appetite can together identify with high specificity and moderate sensitivity carriers of COVID-19. The study also suggests that anosmia and dysgeusia may serve as screening methods for patients with COVID-19 during an epidemic.^21^

Mao et al. identified an incidence of anosmia of 5.1% among patients hospitalized in China with confirmed diagnosis of COVID-19.^11^ The Chinese study only evaluated inpatients, differently from our study where outpatients were analyzed. It is not yet known whether SLoS is a more characteristic symptom in patients with mild or severe Covid-19. This fact highlights that SLoS is not the main symptom in COVID-19 pandemic but once present, patients have near to 90% probability of being infected.

In Iran, Bagheri et al. accessed patients remotely through a questionnaire with no possibility of laboratory confirmation of either case. They observed a linear correlation between the proportion of studied cases of anosmia or hyposmia and positive cases of COVID-19 (*p* < 0.001). In the same study, only 1.1% of the patients who reported altered olfaction were hospitalized for respiratory distress.^4^

Currently, it is not possible to determine if there will be a recovery of the sense of smell and how long this will take to recover.^5^, ^19^ In our study almost 50% of those who were followed up showed improvement in the symptoms of SLoS in two weeks. We also observed that allergic rhinitis comorbidity did not impact the improvement of smell loss.

This study presents some limitations imposed by the context of the COVID-19 pandemic and the very nature of the study. Considering that the data survey was performed using an online instrument, all questionnaires were answered by the patients themselves. This occurred because the cost of interruption of social distance would not be compensated by face-to-face medical care. Questions related to the proper completion of the questionnaire were resolved through direct contact between researchers and participants via electronic messaging and telephone.

Another aspect is related to the methodology used for testing the cases, which was not discriminated, although we know that for much of the period where the data was collected, only RT-PCR testing was available in Brazil. It was not possible to establish a correlation between the severity of the clinical condition and SLoS, once our study was designed to evaluate outpatients.

Finally, our study showed no significant differences between comorbidities associated with SLoS in patients who tested positive or negative for COVID-19, making this symptom an excellent predictor for SARS-CoV-2 infection.

So far, the tests available to diagnose COVID-19 have low sensitivity. Therefore, false-negative results are common. Thus, the PPV of SLoS to diagnose COVID-19 observed in this study (88.8%) is probably underestimated, which leads us to believe that the actual PPV of SLos should be even higher during the COVI-19 pandemic.

The clinical behavior of COVID-19 is quite variable, with no pathognomonic symptom identified at this time. Given the low sensitivity and the scarcity of laboratory tests, SLoS may be useful as diagnostic criteria under epidemic situations, dismissing complementary tests and allowing the institution of immediate measures based on a presumptive diagnosis with high accuracy.

## Conclusion

There is a strong relationship between SLoS and COVID-19, which can be considered a highlighting symptom of the disease during the epidemic, due to its elevated PPV. The identification of SLoS during an epidemic may serve as a sentinel symptom and may be a warning to establish measures to prevent the transmission of the disease. Moreover, in an epidemic of COVID-19, SLoS could be considered as a clinical criterion for COVID-19 diagnosis when laboratory tests are not available.

## Conflicts of interest

The authors declare no conflicts of interest.

## References

[1] Park S.E. (2020). Epidemiology, virology, and clinical features of severe acute respiratory syndrome coronavirus-2 (SARS-CoV-2; Coronavirus Disease-19). Clin Exp Pediatr.

[2] Candido D.D.S., Watts A., Abade L., Kraemer M.U.G., Pybus O.G., Croda J. (2020). Routes for COVID-19 importation in Brazil. J Travel Med.

[3] Boletim Epidemiológico – Secretaria de Vigilância em Saúde; 2020. Available from: https://covid.saude.gov.br/ [cited 11.04.20]

[4] Bagheri S.H.R., Asghari A.M., Farhadi M., Shamshiri A.R., Kabir A., Kamrava S.K. (2020). Coincidence of COVID-19 epidemic and olfactory dysfunction outbreak. medRxiv.

[5] Brann D., Tsukahara T., Weinreb C., Logan D.W., Datta S.R. (2020). Non-neural expression of SARS-CoV-2 entry genes in the olfactory epithelium suggests mechanisms underlying anosmia in COVID-19 patients. bioRxiv.

[6] Li K., Wohlford-Lenane C., Perlman S., Zhao J., Jewell A.K.RL (2016). Middle east respiratory syndrome coronavirus causes multiple organ damage and lethal disease in mice transgenic for human dipeptidyl peptidase 4. J Infect Dis.

[7] Xu K., Lai X.Q., Liu Z. (2020). Suggestions for prevention of 2019 novel coronavirus infection in otolaryngology head and neck surgery medical staff. Zhonghua Er Bi Yan Hou Tou Jing Wai Ke Za Zhi.

[8] Bavishi C., Maddox T.M., Messerli F.H. (2020). Coronavirus disease 2019 (COVID-19) infection and renin angiotensin system blockers. JAMA Cardiol.

[9] Steardo L., Steardo L., Zorec R., Verkhratsky A. (2020). Neuroinfection may potentially contribute to pathophysiology and clinical manifestations of COVID-19. Acta Physiol.

[10] Baig A.M., Khaleeq A., Ali U., Syeda H. (2020). Evidence of the COVID-19 virus targeting the CNS: tissue distribution, host–virus interaction, and proposed neurotropic mechanisms. ACS Chem Neurosci.

[11] Mao L., Wang M., Chen S., He Q., Chang J., Hong C. (2020). Neurological manifestations of hospitalized patients with COVID-19 in Wuhan, China: a retrospective case series study. JAMA Neurol.

[12] Giacomelli A., Pezzati L., Conti F., Bernacchia D., Siano M., Oreni L. (2020). Self-reported olfactory and taste disorders in SARS-CoV-2 patients: a cross-sectional study. Clin Infect Dis.

[13] Wilson R.S., Yu L., Bennett D.A. (2011). Odor identification and mortality in old age. Chem Sens.

[14] Lüers J.-C., Klußmann J.P., Guntinas-Lichius O. (2020). Die Covid-19-Pandemie und das HNO-Fachgebiet: Worauf kommt es aktuell an?. Laryngo-Rhino-Otologie.

[15] Potter M.R., Chen J.H., Lobban N.-S., Doty R.L. (2020). Olfactory dysfunction from acute upper respiratory infections: relationship to season of onset. Int Forum Allergy Rhinol.

[16] Suzuki M., Saito K., Min W.-P., Vladau C., Toida K., Itoh H. (2007). Identification of viruses in patients with postviral olfactory dysfunction. Laryngoscope.

[17] Vukkadala N., Qian Z.J., Holsinger F.C., Patel Z.M., Rosenthal E. (2020). COVID-19 and the otolaryngologist – preliminary evidence-based review. Laryngoscope.

[18] Gupta K., Kumar Mohanty S., Kalra S., Mittal A., Mishra T., Ahuja J. (2020).

[19] Vaira L.A., Salzano G., Deiana G., De Riu G. (2020). Anosmia and ageusia: common findings in COVID-19 patients. Laryngoscope.

[20] Eliezer M., Hautefort C., Hamel A.-L., Verillaud B., Herman P., Houdart E. (2020). Sudden and complete olfactory loss function as a possible symptom of COVID-19. JAMA Otolaryngol Head Neck Surg.

[21] Wang W., Xu Y., Gao R., Lu R., Han K., Wu G. (2020). Detection of SARS-CoV-2 in different types of clinical specimens. JAMA.

[22] Zhao J., Yuan Q., Wang H., Liu W., Liao X., Su Y. (2020). Antibody responses to SARS-CoV-2 in patients of novel coronavirus disease 2019. SSRN Electron J.

[23] Menni C., Valdes A., Freydin M.B., Ganesh S., El-Sayed Moustafa J., Visconti A. (2020). Loss of smell and taste in combination with other symptoms is a strong predictor of COVID-19 infection. medRxiv.

[24] Conseil National Professionel de l’ORL (2020). https://www.snorl.org/category-acces-libre/category-actualites/alerte-anosmie-covid-19-20-mars-2020/.

[25] Hopkins C.K.N. (2020).

[26] AAO-HNS (2020). https://www.entnet.org/content/aao-hns-anosmia-hyposmia-and-dysgeusia-symptoms-coronavirus-disease.

